# Sustained Swimming Training Is Associated With Reversible Filet Texture Changes of European Sea Bass (*Dicentrarchus labrax* L.)

**DOI:** 10.3389/fphys.2019.00725

**Published:** 2019-06-13

**Authors:** C. Shi, J. Wang, Z. Yang, X. Gao, Y. Liu, C. Wang

**Affiliations:** ^1^Key Laboratory of Applied Marine Biotechnology, Ministry of Education, Faculty of Life Science and Biotechnology, Ningbo University, Ningbo, China; ^2^Collaborative Innovation Center for Zhejiang Marine High-Efficiency and Healthy Aquaculture, Ningbo, China; ^3^Institute of Oceanology, Chinese Academy of Sciences, Qingdao, China; ^4^College of Marine Technology and Environment, Dalian Ocean University, Dalian, China

**Keywords:** sustained training, muscle cellularity, growth performance, flesh quality, detraining effect

## Abstract

This present study aimed to investigate the effect of training and detraining on the growth, chemical composition, white muscle fibers, and filet texture of the European sea bass (*Dicentrarchus labrax* L.). Fish were divided into control and training groups, which were subjected to water velocities of 0.2 and 1.0 body length per second (bl s^−1^), respectively, for 32 days (phase I). Half of the fish in the training group were then randomly selected and detrained at a velocity of 0.2 bl s^−1^ for another 32 days (detraining group), while the velocity of the remaining fish in the training group (1 bl s^−1^), and control group (0.2 bl s^−1^) remained unchanged (phase II). The results showed that the growth, body composition, and white muscle fiber densities of the control and trained fish were not significantly different in either phase. Training significantly altered the muscle fiber distribution (*P* < 0.05), with the training group having fewer 80–90 μm fibers than the control and detraining group at the end of the experiment (*P* < 0.05). The training group also had significantly higher values for white fiber muscle textural parameters (hardness, adhesiveness, cohesiveness, springiness, gumminess, and chewiness) in phase I (*P* < 0.05), and these parameters correlated significantly with pH (*P* < 0.05). However, these differences in texture and the pH correlation weakened when the fish were detrained in phase II. These results indicated that an increase in muscle pH after training may alter the flesh texture characteristics of sea bass. In addition, sustained swimming could induce a reversible change in the filet texture of sea bass.

## Introduction

Swimming is an important aspect of the life history of most teleost fish species in aquatic environments. Teleost fish have a great capacity for both sustained (Magnoni et al., 2013; Palstra et al., 2015) and burst swimming (Tudorache et al., 2010; Osachoff et al., 2014), and high water velocities promote the growth and feed conversion efficiency of these fish (Jobling et al., 1993; Davison, 1997). This effect of high water velocity (and sustained swimming under these conditions) is believed to be the result of hyperplasia (the growth of new small fibers) and hypertrophy (the growth of existing fibers) (Li et al., 2016). Thus, changes in white muscle fiber are thought to be the main reason for fish growth (Martin and Johnston, 2005). However, a number of studies found that white muscle fiber changes were not positively correlated with fish growth; however, this could be attributed to differences in species, training protocols, and sampling positions (Martin and Johnston, 2006; Ibarz et al., 2011).

Significant differences in white muscle cellularity between trained and untrained fish have been observed (Davison, 1997; Johnston, 1999; Bugeon et al., 2003; Martin and Johnston, 2005; Ibarz et al., 2011), and the parameters responsible for these differences may be related to flesh quality (Periago et al., 2005). Consequently, sustained swimming of fish could be a practical way to improve the flesh quality (Bugeon et al., 2003). A velocity of 0.8–2 body length per second (bl s^−1^) had a positive effect on the growth and flesh quality of many species (Davison and Goldspink, 1977; Greer and Emerson, 1978; East and Magnan, 1987; and reviewed by Palstra and Planas, 2011). Sustained swimming at a velocity of 1.5 bl s^−1^ improved muscle growth and cellularity in the gilthead sea bream (*Sparus aurata*) (Ibarz et al., 2011). Juvenile qingbo (*Spinibarbus sinensis*) swimming under moderate water velocities exhibited a higher protein content in their muscle, and increased total essential amino acids (ΣEAA) (at 2 bl s^−1^) and total amino acids (ΣAA) (at 1 and 2 bl s^−1^) compared with control fish. However, the lowest levels ΣAA and total n–6 poly unsaturated fatty acids (Σn–6 PUFA) were observed in fish swimming at a water velocity of 4 bl s^−1^ (Li et al., 2016). Moderate water velocities of 1 bl s^−1^ have induced changes in the fatty acid profile and texture of filets in rainbow trout *Oncorhynchus mykiss* (Walbaum) (Rasmussen et al., 2011). The textural characteristics of fish, which are determined not only by muscle cellularity but also by other parameters, such as collagen content and pH, were also influenced by exercise (Johnston, 1999; Johnston et al., 2000; Periago et al., 2005). Post-training elevations in muscle pH may delay denaturalization and enhance the water holding capacity of the sarcoplasmic proteins, consequently changing the tenderness of the flesh (Davie and Sparksman, 1986). Evidence has shown that even relatively low water velocities around 1 bl s^−1^ could induce various physiological effects in salmonids (Johnston et al., 2000; Martin and Johnston, 2005; Rasmussen et al., 2011) and other species, such as kingfish *Seriola lalandi* (25.5% U_crit_, 0.75 bl s^−1^) (Brown et al., 2011) and qingbo (1 bl s^−1^) (Li et al., 2016). Meanwhile, maintaining the velocity at a low level will not decrease the efficiency of biofiltering (low hydraulic retention time) or increase the energy cost of the system. From an economic point of view, it is important to find a velocity that is as low as possible, which at the same time, improves the quality of the fish. However, although training has been found to change the flesh quality (Palstra and Planas, 2011), the duration of these changes is largely unknown, which is important to design a training strategy aimed at improving flesh quality.

The European sea bass *Dicentrarchus labrax* L. is a temperate perciform species that is economically important in the Mediterranean and western Atlantic (Marras et al., 2010). Locomotion has an important role in life cycle of European sea bass; as a result, the individual’s anaerobic burst swimming and sustained aerobic swimming ability could potentially influence its survival, growth, and reproduction performance (Marras et al., 2014). Several studies have examined the aerobic and anaerobic swimming ability of sea bass (Marras et al., 2010, 2014), and assessed the maximal sustainable (critical) swimming speed (U_crit_) (Chatelier et al., 2005). For instance, a velocity of 25.5% U_crit_ (0.75 bl s^−1^) could significantly increase growth in the New Zealand yellowtail kingfish, *S. lalandi*. Sea bass (average mass 501 g; fork length 34 cm) has a U_crit_ of 2.25 bl s^−1^; therefore, it is likely that swimming at 25% of the U_crit_ would improve growth, as seen in yellowtail kingfish. Our hypothesis was that a sustained training protocol at a low velocity of 1 bl s^−1^ would improve the filet texture and growth of fish. In the present study, the fish were trained at a velocity of 1.0 bl s^−1^ (representing about 44% of the U_crit_, calculated according to Chatelier et al., 2005) (1) to determine whether sustained swimming training improves the flesh quality in European sea bass, and (2) to investigate whether sustained swimming training affects white muscle fibers and growth performance (specific growth rate (SGR) and condition factors in this study). Furthermore, the effect of detraining was also studied, which to the best of our knowledge, makes this the first study on the effect of detraining on flesh quality parameters in sea bass.

## Materials and Methods

### Fish Husbandry and the Experimental Protocol

**FIGURE 1:**
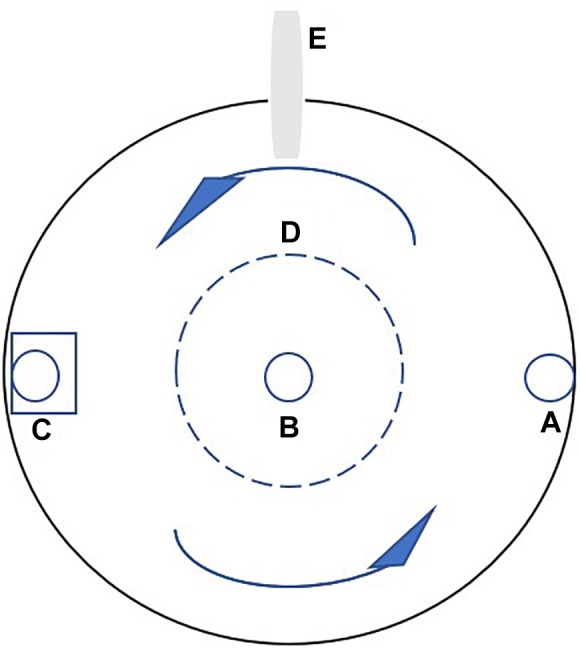
The platform of the experimental tank. **(A)** The water inlet, **(B)** the water outlet, **(C)** the pump with a vertical perforated pipe, **(D)** plastic hoarding, and **(E)** the feeding tube of the self-feeder. The arrows indicate the current direction.

European sea bass (fork length 20.79 ± 0.39 cm, mean ± SD) were obtained from a fish farm in Tianjin, China. Fish of that size were selected because they would probably reach the minimum market size (about 300 g) at the end of the training period; therefore, the training effect on flesh quality would potentially affect the price of the fish. A Passive Integrated Transponder (PIT-tag) was implanted horizontally into each fish, just behind the skull, for individual recognition and calculation of growth performance. The implant did not affect the swimming behavior of the experimental fish, as observed in previous studies (Li et al., 2017). The experiment lasted 64 days and was divided into two stages: Phase I (1–32 days) and phase II (33–64 days). In phase I, the fish were randomly divided into the control and training groups with 30 fish per tank in triplicate. Water velocities of 0.2 and 1.0 bl s^−1^ were applied in the control and training groups, respectively. The water inlet and submerged pump equipped with a vertical perforated pipe were used to adjust the water velocity. A round plastic hoarding with holes and a diameter of 25 cm was placed in the center of the tank to keep fish from entering the low velocity region (Figure 1). Water velocities were measured every week using a flow meter (INFINITY-EM AEM-US, Japan) placed underwater with a depth of 30 cm. The average water velocity at different positions in the tanks differed by less than 15%. In phase II, to study the effect of detraining, the training group was evenly divided into two groups in triplicate: in one group, the water velocity was maintained at 1.0 bl s^−1^ (training group), while the other group was detrained at a velocity of 0.2 bl s^−1^ (detraining group). The velocity of the control group remained the same (0.2 bl s^−1^), while half of the remaining fish (15 individuals) were randomly removed to keep the stocking density identical with training and detraining groups (Table 1). At the end of each phase, the fish were anesthetized using tricaine methanesulfonate (80 mg L^−1^), and their individual weight and length were measured. Four fish were randomly selected from each tank, and euthanized with an overdose of tricaine methanesulfonate (300 mg L^−1^) at each sampling (12 fish were euthanized per group per sampling).

**Table 1:** Experimental protocol and body weights of sampled fish.

	Velocity (bl s^−1^)			Body weight of sampled fish (g)		
	Control	Training	Detraining	Control	Training	Detraining
Phase I	0.2	1.0	1.0	182.03 ± 4.05	195.08 ± 5.53	201.21 ± 9.42
Phase II	0.2	1.0	0.2	292.31 ± 16.22	303.24 ± 10.38	311.93 ± 21.53

*bl s^−1^; body length per second.*

Self-feeders with string triggers (Hishing Electronics Co. Ltd., Qingdao, China) were placed in each tank. The trigger was a black bead (diameter, 5 mm) suspended at the edge of the tank, about 1.5 cm below the water surface. Each activation would deliver 30 pellets (0.45 g/kg fish). A commercial feed (Aller Aqua Co. Ltd., Christiansfeld, Denmark) which contained 47% protein, 22% crude lipid, and 12% ash was used. The self-feeder was connected to a computer and the ration level (RL) was recorded.

Circular tanks of 600 L net water volume (water height: 90 cm, diameter: 90 cm) were used in the experiment (Figure 1). Each tank was equipped with a solid-liquid separator so that the feces and residual feed could be collected. Fish were reared under a 12:12 h (light/dark) cycle. Tanks were supplied with water with high dissolved oxygen (O_2_ ≥ 6.0 mg l^−1^) and a low ammonia concentration (≤0.25 mg l^−1^). The water temperature was maintained at 26 ± 0.5°C and the salinity was maintained between 28 and 30%. The flow rate in each tank was 0.5 m^3^ h^−1^.

### Calculation of Production Parameters

The SGR, condition factor, RL, and feed conversion ratio (FCR) were calculated as follows:

$$\text{SGR}=100\times (\ln\;W_{t}-\ln\;W_{0})/t$$

RL=100×Cw/((Wt+W0)/2×t

$$\mathrm{Condition factor}=100\times (W/L^{3})$$

$$\text{FCR}=C_{w}/(W_{t}-W_{0}).$$

In the equations, *W_t_* and *W*_0_ are the final and initial wet body weight (g) of the fish, respectively, *t* is the feeding duration (days), *C_w_* is the feed intake in terms of weight (g), *L* is the total length (mm), and *W* is the weight of the fish (g).

### Histological Analyses

Muscle tissue blocks (1.5 × 1.5 cm) were obtained from the left filet just under the dorsal fin. Blocks were placed in optimal cutting compound (SAKURA, Torrance, CA, United States), transferred to liquid nitrogen and stored at −80°C before sectioning. Sections of 8-μm thickness were obtained using a cryostat (Leica CM1950) and stained using hematoxylin and eosin (Stickland, 1983). The sections were photographed under a light photomicroscope (NiKon80i), and morphometric analysis was carried out using Image pro Plus 6.0. Fibers (200–400 per fish) were analyzed for their cross-sectional area. The diameter (*d* = 2r) of each fiber was calculated from the fiber area (A) [*A* = π ⋅ r^2^→*d* = 2√A π^−1^)] and the circularity of each fiber (4π fiber area circumference^−2^). A circularity of 1.0 indicated a complete circle (Rasmussen and Ostenfeld, 2000). For each fish, the diameters of at least 400 white muscle fibers were measured to calculate the fiber density (number of muscle fibers per mm^2^) (Johnston et al., 2000).

### Textural Parameters

The right filet was dissected from the fish. The textural parameters of the dorsal muscle were measured using a Texturometer (TMS-PRO, Food Technology Corporation, Sterling, VA, United States) with a 25 N load cell, an 8-mm spherical probe, and a test speed of 30 mm/min. Each sample was measured four times in the dorsal muscle, perpendicular to the muscle fiber orientation. Textural parameters including hardness (peak force of the first compression cycle), adhesiveness (the negative force area of the first compression cycle represented as the work necessary to pull the compressing plunger away from the sample), cohesiveness (the ratio of the positive force areas under the first and second compressions cycles), springiness (the distance by which the food recovered its height during the time that elapsed between the end of the first compression cycle and the start of the second compression cycle), gumminess (the product of hardness and cohesiveness) and chewiness (the product of springiness and gumminess) were calculated using the distance, maximum force, and maximum shear force values obtained from the texture profile curve of each sample (Shao et al., 2016).

### Chemical Analyses

The feed and filet were oven dried at 75°C to a constant weight to determine the moisture content. The nitrogen contents of the feed and filet were measured using a Vario EL III Elemental Analyzer (Elementar, Dortmund, Germany). The energy content was measured using a calorimeter (PARR Instrument Company, Moline, IL, United States). Proximate compositions were analyzed using a standard procedure (Association of Official Analytical Chemists [AOAC], 1990). Filet samples (about 10 g) were homogenized in distilled water at a ratio of 1:5 (weight:volume) using a Pro2000 homogenizer (PRO Scientific Oxford, CT, United States). The pH values of the homogenates were immediately measured using a portable pH meter (Testo 206, Testo AG Company, Lenzkirch, Germany). The pH measurements were performed in triplicate and expressed as an average (Periago et al., 2005). The collagen and hydroxyproline contents were measured after acid hydrolysis of the flesh filet and spectrophotometric measurement of the compound formed from *p*-dimethylaminobenzaldehyde (Periago et al., 2005).

### Statistical Analyses

Data were analyzed using SPSS for Windows (Version 21.0, IBN, Armonk, NY, United States). Before analysis, raw data were analyzed using the Kolmogorov–Smirnov test for normality of distribution and homogeneity of variance. Non-normal and heterogeneous data were transformed using logarithmic or square root transformation until normality and homogeneity were achieved. ANCOVA (analysis of covariance) was applied to analyze the filet components, textural parameters, and muscle cellularity. Water velocity was fitted as a fixed effect, and the final weight of the individuals was fitted as a covariate. The growth parameters, including SGR, body weight, size heterogeneity, condition factor, RL, and FCR were compared using Student’s *t*-test in phase I and using Tukey’s multiple comparison *post hoc* test in phase II. Pearson correlation analyses were carried out to detect any correlations between the analyzed parameters. There was no difference in any of the parameters between tanks in the same treatment, and as a result, further statistical analysis on the tanks was excluded.

## Results

### Growth Parameters and Distribution of Fiber Size

**FIGURE 2:**
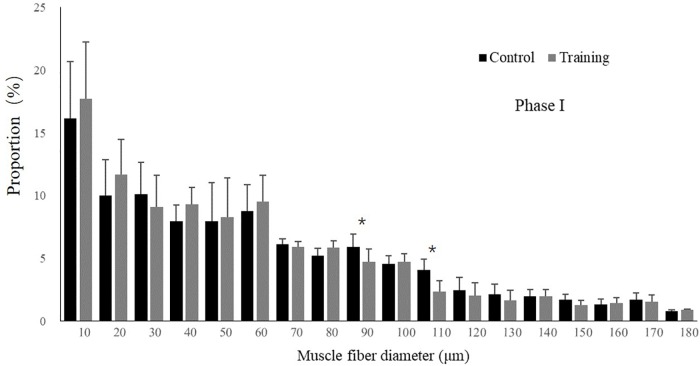
Distribution of fiber size in the control and training groups (mean ± SD) in Phase I (*n* = 12). ^∗^Significant differences between the experimental groups (*P* < 0.05).

**FIGURE 3:**
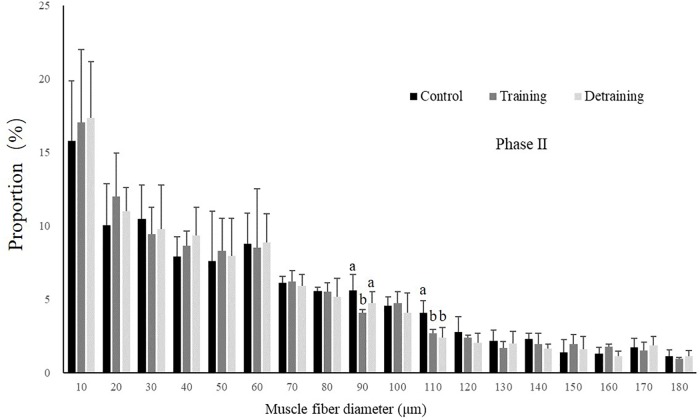
Distribution of fiber size in the control, training, and detraining groups (mean ± SD) in Phase II (*n* = 12). Different letters indicate significant differences in the same muscle fiber diameter range between the experimental groups (*P* < 0.05).

No mortality occurred during the experiment. There was no significant difference in the condition factor, RL, and FCR between the groups in both experimental stages (Table 2). In phase II, the training group had slightly higher SGR than the control group and the detraining group; however, the difference did not reach statistical significance. The general fiber size distribution differed significantly between the control and training group (*P* < 0.05) in both phases; however, no significant difference was observed between the training and detraining group in phase II (Figure 2, 3). The average fiber diameters did not differ significantly between the three groups in phase II (Table 3). In phase II, the control group still had more 100–110 μm fibers than the training and detraining group. However, the detraining group had a similar proportion of 80–90 μm fibers to the control group, and had a significantly higher proportion of these fibers compared with that in the training group (*P* < 0.05) (Figure 3).

**Table 2:** SGR, initial and final body weight, condition factor, ration level (RL), and FCR of control and exercised sea bass (mean ± SD) (three replicates with 30 fish per tank in Phase I and 26 fish in Phase II).

Parameters	Phase I		Phase II		
	Control	Training	Control	Training	Detraining
SGR (% d^−1^)	1.42 ± 0.11	1.51 ± 0.13	1.56 ± 0.19	1.72 ± 0.21	1.62 ± 0.17
Initial body weight (g)	112.44 ± 11.61	116.97 ± 15.75	172.09 ± 15.05	180.42 ± 16.73	184.42 ± 16.73
Final body weight (g)	177.06 ± 14.07	189.75 ± 15.74	284.05 ± 18.39	313.33 ± 19.21	309.33 ± 19.21
Initial condition factor	1.26 ± 0.03	1.30 ± 0.02	1.34 ± 0.03	1.36 ± 0.06	1.37 ± 0.06
Final condition factor	1.32 ± 0.02	1.39 ± 0.05	1.34 ± 0.04	1.40 ± 0.13	1.42 ± 0.13
Ration level (% bw d^−1^)	2.54 ± 0.76	2.56 ± 0.32	2.72. ± 0.41	2.74 ± 0.39	2.81 ± 0.28
FCR	1.83 ± 0.68	1.74 ± 0.29	1.90 ± 0.58	1.61 ± 0.23	1.64 ± 0.23

*SGR, specific growth rate; FCR, feed conversion ratio; bw d^−1^, body weight per day.*

**Table 3:** Physicochemical and muscle cellularity of the flesh of different groups (mean ± SD) (*n* = 12).

Parameters	Phase I (0–32 days)		Phase II (32–64 days)		
	Control	Training	Control	Training	Detraining
Crude protein (%)	17.73 ± 1.14	18.40 ± 0.98	17.47 ± 1.14	17.32 ± 0.97	18.58 ± 0.55
Crude lipid (%)	9.23 ± 1.55	9.57 ± 0.82	9.36 ± 1.54	8.73 ± 2.12	9.49 ± 2.03
Ash (%)	4.10 ± 0.30	4.36 ± 0.21	4.74 ± 1.04	4.28 ± 1.11	4.36 ± 0.31
Moisture (%)	68.43 ± 2.42	66.37 ± 2.15	69.81 ± 2.73	67.88 ± 2.72	67.93 ± 2.83
Energy (kJ g^−1^)	12.47 ± 0.98	12.09 ± 1.25	10.10 ± 1.31	10.15 ± 2.01	11.15 ± 0.80
Hydroxyproline (μg/g)	1170.49 ± 83.81	1263.48 ± 102.99	1202.93 ± 83.81	1271.39 ± 91.24	1255.68 ± 116.16
Collagen (%)	0.26 ± 0.11	0.32 ± 0.02	0.31 ± 0.02	0.34 ± 0.04	0.35 ± 0.05
Collagen/Total protein (%)	1.49 ± 0.68	1.76 ± 0.16	1.76 ± 0.12	1.92 ± 0.22	1.88 ± 0.24
pH	6.61 ± 0.08	6.75 ± 0.10^∗^	6.55 ± 0.16	6.78 ± 0.14^∗^	6.66 ± 0.09
**Muscle cellularity**					
White muscle fiber diameter (μm)	84.50 ± 3.88	79.16 ± 2.14	89.67 ± 11.89	84.75 ± 12.83	83.00 ± 9.05
White muscle fiber density (number per mm^2^)	179.92 ± 17.31	192.69 ± 12.88	168.80 ± 9.50	171.80 ± 17.33	177.24 ± 16.22

*^∗^Significant difference (P < 0.05).*

### Physicochemical Parameters of the Flesh

**FIGURE 4:**
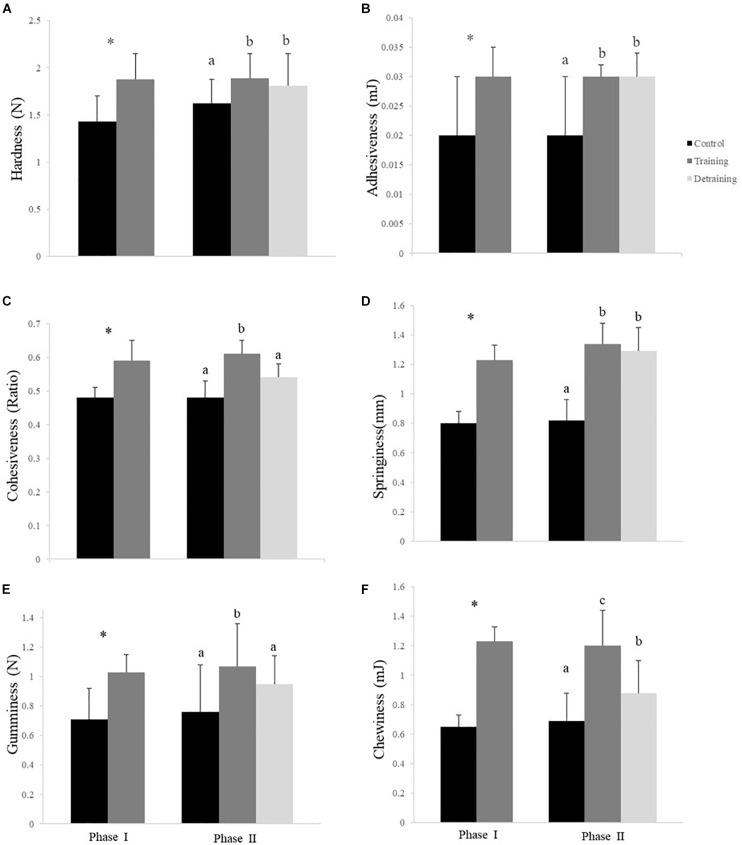
Hardness **(A)**, adhesiveness **(B)**, cohesiveness **(C)**, springiness **(D)**, gumminess **(E)**, and chewiness **(F)** (mean ± SD) in the flesh of the experimental fish (*n* = 12). ^∗^Significant differences between the control and training groups in Phase I; different letters indicate significant differences among control, training, and detraining groups in Phase II (*P* < 0.05).

The composition of the filets barely differed between the groups in both phases, with no significant differences observed in the protein, lipid, ash, moisture, and energy contents (Table 3). The hydroxyproline, collagen, and collagen/total protein contents were not affected by the training protocol (Table 3). The training group showed a significantly higher pH over the entire experimental period (*P* < 0.05); in phase II, when the training was discontinued, the pH in the detraining group decreased to levels similar to that in the control group (*P* > 0.05) (Table 3). At the end of phase I, the training group had significantly higher textural parameter values than the control group, including hardness (1.88 ± 0.27 *vs*. 1.43 ± 0.27), adhesiveness (0.03 ± 0.00 *vs*. 0.02 ± 0.01), cohesiveness (0.59 ± 0.06 *vs*. 0.48 ± 0.03), springiness (1.23 ± 0.14 *vs*. 0.80 ± 0.16), and gumminess (1.03 ± 0.12 *vs*. 0.71 ± 0.21) (Figure 4). In phase II, the training group had higher textural parameter values than the control group and higher cohesiveness, gumminess, and chewiness than the detraining group (*P* < 0.05); however, there was no significant difference in hardness, adhesiveness, and springiness between the training and detraining fish filets (Figure 4).

### Correlation Between Muscle Cellularity, pH, and Textural Parameters

Table 4 shows the correlations between textural parameters, muscle fiber density, and pH in phase I and phase II. In phase I, the pH values were closely related to textural parameters (*P* < 0.05) (Table 4), while in phase II, only hardness correlated with pH. The muscle fiber density did not correlate with textural parameters over the entire experimental period (Table 4).

**Table 4:** Correlation coefficients and significance levels for muscle cellularity, pH, and textural parameters of sea bass.

	Phase I				Phase II			
	White muscle fiber density		pH		White muscle fiber density		pH	
	Correlation	Sig.	Correlation	Sig.	Correlation	Sig.	Correlation	Sig.
**Textural parameters**								
Hardness (N)	0.271	0.211	0.522	0.014^∗^	0.192	0.231	0.516	0.043^∗^
Adhesiveness (mJ)	0.254	0.232	0.431	0.031^∗^	0.245	0.325	0.273	0.144
Cohesiveness (ratio)	0.381	0.078	0.522	0.006^∗^	0.246	0.397	0.296	0.161
Springiness (mm)	0.286	0.094	0.503	0.029^∗^	0.324	0.431	0.279	0.149
Gumminess (N)	0.275	0.143	0.534	0.022^∗^	0.245	0.452	0.282	0.134
Chewiness (mJ)	0.362	0.112	0.588	0.013^∗^	0.311	0.463	0.265	0.153

*Sig., significance; ^∗^significant correlation.*

## Discussion

The present study demonstrated that 32 days of training did not significantly affect growth, body composition, and white muscle fiber densities compared with those of the control fish. However, the muscle fiber distribution was significantly altered, with the training group having fewer 80–90 μm fibers compared with those in the control and detraining groups. Furthermore, the results showed an effect of training on white muscle fiber textural parameters, which correlated positively with pH. However, these correlations diminished after detraining. Thus, although training changed white muscle fiber textural parameters, and this was correlated with an increase in muscle pH, these changes disappeared upon detraining.

Long-term swimming training of fish affects growth and development in many species (Castro et al., 2011; Rasmussen et al., 2011; Graziano et al., 2018). U_opt_ is defined according to the relationships between oxygen consumption and swimming speed, which is typically exponential (Brett, 1964; Webb, 1975; Thorarensen et al., 1993; Gallaugher et al., 2001). Sustained swimming exercise at U_opt_ altered the energy allocation and delayed the testicular development of male European sea bass, as evidenced by a decreased gonadosomatic index and slower progression of testicular development (Graziano et al., 2018). For Atlantic salmon (*Salmo salar* L.) under aerobic exercise (1.06 bl s^−1^), higher activity costs were associated with increased utilization of nutrients and energy for growth (Grisdale-Helland et al., 2013). By contrast, the effects of detraining are relatively less well studied. For the striped bass, *Morone saxatilis*, trained at 2.4–3.6 bl s^−1^ for 60 days, the SGR quickly increased once the training stopped, and the effect on growth persisted for 56 days after training (Young and Cech, 1994). A possible explanation is that during the sustained aerobic training phase, the fish ingest more food than usual and/or increase the utilization of nutrients for growth (Grisdale-Helland et al., 2013). When training is discontinued, they continue to consume as much food and/or maintain the high utilization rate of nutrients. However, such an effect was not observed in the present study: Training and detraining only slightly changed the RL and FCR. The possible reason is that the present training level (at about 44% U_crit_) is too weak to modify the appetite and the energy allocation of the European sea bass of the present size. Although a velocity of 25.5% U_crit_ (0.75 bl s^−1^) could yield a significant growth increase for the New Zealand yellowtail kingfish *S. lalandi*, for the European sea bass, the fish trained at U_opt_ (4.4–6.5 bl s^−1^, <69% U_crit_) did not exhibit significantly different growth rates compared with those of the control groups (Graziano et al., 2018). However, the purpose of using 69% U_crit_ in the training protocol was to delay the testicular development of male European sea bass (Graziano et al., 2018) rather than improve the growth rate. In practice, a relatively high velocity may impair the nutrition and growth, as revealed in qingbo (Li et al., 2016). Thus, a velocity between 44 and 69% U_crit_ should probably be tested to improve the growth of European sea bass.

Training has been reported to increase the diameter of the white muscle fibers in the leopard shark *Triakis semifasciata* (Gruber and Dickson, 1997), rainbow trout (Martin and Johnston, 2005), and gilt head sea bream (Ibarz et al., 2011). White muscle hypertrophy is regarded as the main reason for muscle growth, and the effect of training on muscle size is largely dependent on the training protocol and the size of the fish. Water velocities below 1 bl s^−1^ seem to be at the lower end of the range required to induce changes in muscle structure (Bugeon et al., 2003). Moreover, evidence shows that the minimum velocity at which changes in white muscle fibers are observed is 1.35 bl s^−1^ in larger rainbow trout (>40 cm in length; Wilson and Egginton, 1994). In the present study, sustained training with medium duration only slightly altered the muscle fiber density or diameter in sea bass; thus, in this fish, the muscle fiber may not have been completely recruited in this training protocol. Nevertheless, the muscle fiber distribution in the training and detraining group was significantly different from that in the control group, indicating that other training strategies might induce stronger responses. This result was partly supported by the study of Rasmussen et al., in which a similar protocol was used for rainbow trout (0.9 bl s^−1^ for 8 weeks).

The texture of fish muscle is generally assessed based on textural mechanical properties, which are affected by environmental and intrinsic parameters, such as velocity, nutrition, life stage, and genotype. In the present study, the textural parameter values were significantly higher in the training group in both phases; however, the effect was weakened when the fish were detrained. The lipid and connective tissue contents are important factors that affect textural mechanical properties; however, lipid deposition must be substantial to produce a significant effect (Rasmussen, 2001). In the present study, the lipid content did not change significantly with training; therefore, the effect on lipid deposition was probably not sufficient to produce any related changes in texture. Moreover, in this study, the hydroxyproline and collagen contents were not affected by the training protocol. A positive correlation has been reported between flesh hardness and fiber density in some fish species (Johnston et al., 2000), including the sea bass (Periago et al., 2005); however, in the present study, no correlation was found between muscle fiber density and texture. The changes in texture characteristics could be attributed mainly to the higher pH of the flesh in the training fish compared with that of the control fish. When the fish were detrained, the pH difference between the control and detraining groups vanished, as did the difference in cohesiveness and gumminess. A significant correlation was found between pH and texture in phase I. Tenderization of flesh is caused by enzymatic degradation of myofibrils, intermediate filaments, and cytoskeletal muscle proteins (Koohmaraie, 1996). The sarcoplasm of muscle constitutes approximately 30% of the total protein in muscle. Sarcoplasmic proteins are water-soluble and predominantly include glycolytic enzymes, creatine kinase, and myoglobin (Bowker et al., 2014). Although the relationship between sarcoplasmic proteins and muscle tenderness has not been widely explored because they lack a structural role in muscle (Bowker et al., 2014), the composition of the sarcoplasmic protein fraction isolated from muscle was observed to change with post-mortem aging (Bowker et al., 2008, 2012; Laville et al., 2009). Rapid pH reduction can cause denaturation of the protein, leading to changes in tenderness. By contrast, at the isoelectric point of the protein, the flesh has the lowest water holding capacity. Raising the pH beyond the isoelectric point of the protein causes an imbalance in charge and repulsion of proteins, leading to a better water holding capacity. In this study, increases in muscle pH after continuous training probably delayed the denaturation of the sarcoplasmic proteins and enhanced the water holding capacity, consequently altering the tenderness of the flesh (Delbarre-Ladrat et al., 2004). Similar results were observed in juvenile qingbo subjected to 1–4 bl s^−1^ water velocity, after which they showed higher muscle pH and texture-related parameters (Li et al., 2016). In the present study, the decrease in the textural parameter values in the detrained fish might indicate that the effect of training on muscle texture is reversible and is sustained for less than a month.

## Conclusion

In conclusion, the findings of the present study showed that sustained swimming training (1.0 bl s^−1^) resulted in minor changes in the white fiber density, chemical composition, and growth performance of European sea bass. The trained fish significantly improved the textural characteristics of the filet by causing pH variations; however, the effect lasted for less than 1 month after training was discontinued. Thus, sustained moderate swimming exercise might have a slight, but positive, effect on flesh quality in the European sea bass. A higher water velocity should be investigated in a future study to achieve more significant effects.

## Ethics Statement

The experiments were examined and approved by Ningbo University Institutional Animal Care and Use Committee, to make sure that the procedures conformed to biosecurity and institutional safety regulations.

## Author Contributions

CS carried out the experiment with JW and ZY. XG helped in sampling. YL designed the experiment. CW helped to draft the manuscript.

## Conflict of Interest Statement

The authors declare that the research was conducted in the absence of any commercial or financial relationships that could be construed as a potential conflict of interest.
